# Metabolic Engineering of Non-carotenoid-Producing Yeast *Yarrowia lipolytica* for the Biosynthesis of Zeaxanthin

**DOI:** 10.3389/fmicb.2021.699235

**Published:** 2021-10-07

**Authors:** Yuxiao Xie, Shulin Chen, Xiaochao Xiong

**Affiliations:** Department of Biological Systems Engineering, Washington State University, Pullman, WA, United States

**Keywords:** *Yarrowia lipolytica*, metabolic engineering, zeaxanthin, CrtZ, lycopene, β-carotene

## Abstract

Zeaxanthin is vital to human health; thus, its production has received much attention, and it is also an essential precursor for the biosynthesis of other critical carotenoids such as astaxanthin and crocetin. *Yarrowia lipolytica* is one of the most intensively studied non-conventional yeasts and has been genetically engineered as a cell factory to produce carotenoids such as lycopene and β-carotene. However, zeaxanthin production by *Y. lipolytica* has not been well investigated. To fill this gap, β-carotene biosynthesis pathway has been first constructed in this study by the expression of genes, including *crtE*, *crtB*, *crtI*, and *carRP*. Three *crtZ* genes encoding β-carotene hydroxylase from different organisms were individually introduced into β-carotene-producing *Y. lipolytica* to evaluate their performance for producing zeaxanthin. The expression of *crtZ* from the bacterium *Pantoea ananatis* (formerly *Erwinia uredovora, Eu-crtZ*) resulted in the highest zeaxanthin titer and content on the basis of dry cell weight (DCW). After verifying the function of *Eu-crtZ* for producing zeaxanthin, the high-copy-number integration into the ribosomal DNA of *Y. lipolytica* led to a 4.02-fold increase in the titer of zeaxanthin and a 721% increase in the content of zeaxanthin. The highest zeaxanthin titer achieved 21.98 ± 1.80 mg/L by the strain grown on a yeast extract peptone dextrose (YPD)–rich medium. In contrast, the highest content of DCW reached 3.20 ± 0.11 mg/g using a synthetic yeast nitrogen base (YNB) medium to culture the cells. Over 18.0 g/L of citric acid was detected in the supernatant of the YPD medium at the end of cultivation. Furthermore, the zeaxanthin-producing strains still accumulated a large amount of lycopene and β-carotene. The results demonstrated the potential of a cell factory for zeaxanthin biosynthesis and opened up an avenue to engineer this host for the overproduction of carotenoids.

## Introduction

Zeaxanthin is a C40 hydroxyl-carotenoid with a chemical structure that has an unsaturated polyene chain as a skeleton and two β-rings on each side of the skeleton with a hydroxyl-group located on the 3′ site of each ring. The dietary intake of zeaxanthin is vital to human health since zeaxanthin cannot be synthesized in human bodies (Ribaya-Mercado and Blumberg, 2004). Zeaxanthin is found in the human eyes, liver, kidneys, ovaries, and other organs. The conjugated double bonds of lutein provide a substantial antioxidant property (Hsu et al., 2011). It was reported that zeaxanthin could potentially inhibit the invasion and metastasis of several types of cancer cells (Terasaki et al., 2014). Zeaxanthin and its isomer, lutein, provide unique health benefits to the human macular region. Only these two carotenoids, including zeaxanthin and its isomer, lutein, greatly enrich the human retinal macula region to improve visual function, filter blue light, and prevent oxidative damage (Carpentier et al., 2009). These beneficial effects of zeaxanthin on human health led to the tremendous commercial value of zeaxanthin production.

Traditionally, zeaxanthin has been produced by either chemical synthesis or extraction from plants. The procedure for the chemical synthesis of zeaxanthin was costly and complex, and the synthesized product showed poor activity (Cataldo Von Bohle et al., 2016). The extraction of zeaxanthin from vegetables and fruits suffered from a low product yield and environmental pollution due to the use of large amounts of organic solvents (Hsu et al., 2011; Liau et al., 2011). The microbial production of zeaxanthin offers an alternative and sustainable route for the production of zeaxanthin. The biosynthesis of zeaxanthin in engineered microorganisms provides additional advantages, such as high product yield, a low cost of materials, and low environmental impact. As a result, the model organisms such as *Escherichia coli* and *Saccharomyces cerevisiae* have been metabolically engineered to produce zeaxanthin (Albrecht et al., 1999; Matthews and Wurtzel, 2000; Nishizaki et al., 2007; Sun et al., 2012; Liang et al., 2013).

**FIGURE 1:**
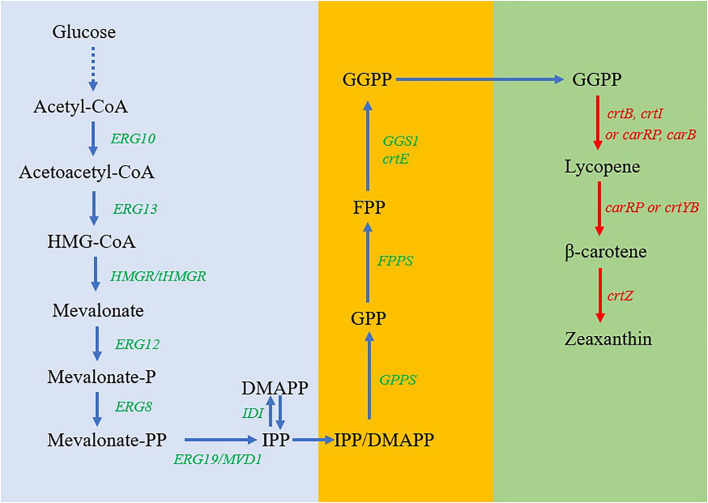
Pathway design and engineering for the biosynthesis of zeaxanthin in *Y. lipolytica*. Blue arrows, native mevalonate (MVA) metabolic pathways in *Y. lipolytica*; red arrows, heterologous genes to be introduced into *Y. lipolytica*; dashed lines, multiple steps for the generation of acetyl-CoA from glucose; accent blue frame, a strategy to enhance the production of IPP; orange frame, a strategy to enhance the production of FPP and GGPP; and green frame, a strategy to enhance the production of zeaxanthin. Metabolites: HMG-CoA, 3-hydroxy-3-methylglutaryl-CoA; IPP, isopentenyl diphosphate; DMAPP, dimethylallyl diphosphate; GPP, geranyl diphosphate; FPP, farnesyl diphosphate; GGPP, geranylgeranyl diphosphate. Enzymes: ERG10, acetyl-CoA thiolase; ERG13, HMG-CoA synthase; ERG12, mevalonate kinase; ERG8, phosphomevalonate kinase; ERG19/MVD1, mevalonate diphosphate decarboxylase; IDI, IPP isomerase; GPS, geranyl phosphate synthase; FPPS, farnesyl pyrophosphate synthase; GGS1 or CrtE, geranylgeranyl diphosphate synthase; CrtB, phytoene synthase; CrtI, lycopene synthase; CarRP, phytoene synthase/lycopene cyclase; CarB, phytoene dehydrogenase; CrtYB, phytoene synthase and lycopene cyclase; CrtZ, β-carotene hydrolase.

Compared with *E. coli* and *S. cerevisiae*, oleaginous yeasts naturally accumulate a large amount of lipids (Ferreira et al., 2018), and the carbon flux can be potentially redirected from lipid biosynthesis toward carotenoids formation (Matthäus et al., 2014). *Yarrowia lipolytica* with Generally Recognized as Safe (GRAS) status is one of the most intensively studied oleaginous yeasts (EFSA Panel on Nutrition, Novel Foods and Food Allergens (Nda), Allergens et al., 2019). Considerable systems biology analyses and well-developed molecular biology tools, including CRISPR/Cas9-based genome editing approach (Abdel-Mawgoud and Stephanopoulos, 2020), have facilitated the systematic metabolic engineering of *Y. lipolytica* for biofuels and renewable chemical production (Worland et al., 2020). *Y. lipolytica* has been engineered as an emerging cell factory for the production of an array of renewable chemicals such as fatty acid (Ghogare et al., 2020), succinic acid (Cui et al., 2017), and triacetic acid lactone (TAL) (Markham et al., 2018). As a well-studied oleaginous yeast strain, *Y. lipolytica* exhibits several advantages as an emerging host for the biosynthesis of carotenoids. First, *Y. lipolytica* can synthesize a massive amount of acetyl-CoA as a precursor of the mevalonate (MVA) pathway, which can provide more geranyl diphosphate (GPP), farnesyl diphosphate (FPP), and geranylgeranyl diphosphate (GGPP) as the intermediates for the accumulation of carotenoids (Czajka et al., 2018; Figure 1). Second, the biosynthesis pathways of β-carotene and astaxanthin have been engineered in *Y. lipolytica* strains (Gao et al., 2017; Kildegaard et al., 2017; Larroude et al., 2018). Third, lipid droplets consisting of large amounts of accumulated oil in the yeast cells can potentially store lipophilic carotenoids (Matthäus et al., 2014). Thus, *Y. lipolytica* has the unique traits as a microbial host for the biosynthesis of carotenoids including zeaxanthin through metabolic engineering, and reconstitution of zeaxanthin biosynthesis can potentially advance our knowledge to understand the mechanisms of carotenoid accumulation, degradation, and sequestration.

Although *Y. lipolytica* does not naturally produce carotenoids, the intermediate GGPP synthesized *via* the MVA pathway can be used for carotenoid biosynthesis with the engineered pathways (Figure 1). In the present study, to allow the strain to produce lycopene and β-carotene, we cloned and expressed *crtE* encoding GGPP synthase, *crtB*, *crtI*, and *CarRP* genes in *Y. lipolytica*. To further extend the pathway for the biosynthesis of zeaxanthin, three different *crtZ* genes encoding β-carotene hydroxylase with optimized codons were individually expressed in *Y. lipolytica* (Figure 1). After identifying the ideal gene based on the zeaxanthin production performance of the recombinants, multiple copies of *crtZ* have been integrated into the genome of *Y. lipolytica* to enhance zeaxanthin production. Finally, production of carotenoids and secretion of citric acid by the engineered strains were investigated in the shake-flask cultures under different cultivation conditions. The results obtained in our studies can provide insight for further improving zeaxanthin production by the optimization of this multiple-gene pathway in *Y. lipolytica*.

## Materials and Methods

### Development of Lycopene-Producing Recombinant Yeast

The auxotrophic strain *Y. lipolytica* PO1f (ATCC MYA-2613) was used as a microbial host for metabolic engineering. The culture conditions for the strains and DNA techniques could be found in our previous publications (Wang et al., 2016; Ghogare et al., 2020; Xiong and Chen, 2020). The episome plasmid pJN44 bearing a constitutive promoter with the first intron, P*_*TEF*1*N*_*, was used to express the genes responsible for producing lycopene. The 0.98-kb native gene *crtE* (YALI0D17050g) encoding GGPP synthase was amplified by PCR with primers CrtE-exp1 and CrtE-exp2 and the genomic DNA of *Y. lipolytica* as a template and then inserted into the expression vector pJN44 after the digestion of the PCR products with the restriction enzymes *Hin*dIII and *Sma*I (Supplementary Table 1). The new plasmid-bearing *crtE* was designated as pJN44-crtE. The codon-optimized genes *crtB* and *crtI* from the bacterium *Pantoea ananatis* were cloned into pJN44 to form the resultant vectors pJN44-crtB and pJN44-crtI, respectively (Matthäus et al., 2014).

The expression cassette of *crtB* containing both the promoter P*_*TEF*1*N*_* and terminator of *xpr2* was excised from the plasmid pJN44-crtB by digestion with the restriction endonucleases *Xba*I and *Spe*I and inserted into the restriction site of *Spe*I of plasmid pJN44-crtE. The two genes *crtE* and *crtB* were co-expressed in the resultant plasmid pCrtEB. Similarly, the expression cassette of gene *crtI* was assembled into plasmid pCrtEB, and a new vector pCrtEBI was constructed to express these three genes, including *crtE*, *crtB*, and *crtI*. *Y. lipolytica* PO1f was transformed with plasmid pCrtEBI. After verifying the function of *Y. lipolytica* transformant to produce lycopene, the selection marker *leu2* and *Y. lipolytica*–replicable region in the plasmid pCrtEBI was replaced with a new selection marker *ura3* flanked with *loxp* sites to form a plasmid pSX23. The plasmid pSX23 was an integrative vector bearing all the three necessary genes for producing lycopene. *Y. lipolytica* PO1f was transformed with linearized pSX23 by the digestion of *Xba*I, and the resultant strain *Y. lipolytica* EBI was tested for lycopene production. After transforming the yeast with the plasmid pJN44-cre expressing *Cre* recombinase, the marker *ura3* in the strain *Y. lipolytica* EBI was removed (Wang et al., 2016; Ghogare et al., 2020). The strain was used for next-step engineering for the biosynthesis of β-carotene.

### Development of β-Carotene-Producing Recombinant Yeast

The 1.84-kb gene *carRP* from *Mucor circinelloides* encoding a lycopene cyclase and phytoene synthase bifunctional enzyme was synthesized by Gene Universal, Inc., (Newark, DE, United States) based on the codon preference of *Y. lipolytica* (Larroude et al., 2018). The DNA sequences of *carRP* were provided in Supplementary Material (Supplementary Table 2). Gene *carRP* was inserted into the expression vector pJN44 in the restriction sites of *Hin*dIII and *Sma*I to form the vector pJN44-CarRP. The plasmid pJN44-CarRP was used to transform lycopene-producing *Y. lipolytica* EBI to test the production of β-carotene. After verifying the function of the *Y. lipolytica* transformant to produce β-carotene, the selection marker *leu2* and *Y. lipolytica*–replicable region in the plasmid pJN44-CarRP was replaced with the selection marker *ura3* flanked with *loxp* sites. The β-carotene-producing strain *Y. lipolytica* CarRP was developed by integrating the expression cassette of *carRP* into the yeast genome with the transformation of the linearized plasmid. The marker *ura3* in *Y. lipolytica* CarRP was removed as described before.

### Development of Zeaxanthin-Producing Recombinant Yeast

The three genes, including *Bv-CrtZ* from *Brevundimonas vesicularis*, *Eu-crtZ* from *P*. *ananatis* (formerly *Erwinia uredovora*), and *Hp-crtZ* from a microalgal strain *Haematococcus lacustris* (Tramontin et al., 2019), were synthesized with the optimized codons for *Y. lipolytica* by Gene Universal, Inc., (Newark, DE, United States). The DNA sequences could be found in Supplementary Material (Supplementary Tables 3–5). These three *crtZ* genes were individually cloned into the plasmid pJN44 in the restriction sites of *Hin*dIII and *Sma*I. The expression vectors pBv-crtZ, pEu-crtZ, and pHp-crtZ were obtained and were used to transform β-carotene-producing yeast. A plasmid pZX13 containing a portion of the ribosomal DNA (rDNA) of *Y. lipolytica* and the *ura3* selection marker was developed for the multiple-copy integration of a targeted gene into the yeast genome (Lv et al., 2019). The schematic map of plasmid pZX13 was shown in Supplementary Figure 1. The expression cassette of *Eu-crtZ* was excised from the plasmid pEu-crtZ and cloned into pZX13. The resultant plasmid was designated as pCV47. The high-copy-number integration of *Eu-crtZ* into the rDNA region of *Y. lipolytica* was achieved by the transformation of *Y. lipolytica* CarRP with linearized plasmid pCV47.

### Strains Cultivation for Producing Carotenoids

The seed cultures were made by transferring single colonies from an agar plate to a shake flask containing 20 ml media and then incubating at 28°C and a shaking speed of 200 rpm for 48 h. We then used the seed cultures to inoculate 50 ml of culture media in a 250-ml baffled flask, and the initial absorbance at 600 nm (OD_600_) of the culture was adjusted to 0.05–0.1. Two media were used for the production of zeaxanthin. The rich medium was a YPD medium consisting of 10 g/L of yeast extract (Difco), 20 g/L of peptone (Difco), and 50 g/L of glucose. The synthetic medium was made with 50 g/L glucose, 6.7 g/L yeast nitrogen base (YNB) (United States Biological, Salem, MA, United States), and 2.0 g/L of the supplement of amino acids (United States Biological, Salem, MA, United States).

### Analysis of Carotenoids Contents

Half a milliliter of cells were harvested from the culture and transferred into 2 ml centrifuge tubes with a screwable cap. After centrifuging at 10,000 rpm for 2 min, the supernatant was removed from the tube. The same volume of 0.5 mm glass beads was added into the tubes together with 1 ml of ethyl-acetate. After placing the tubes on ice for 5 min, the yeast cell wall was disrupted by a cell disruptor, FastPrep FP120. After centrifuging the tube at 10,000 rpm for 2 min, the supernatant was collected and transferred into new 2 ml tubes. The extraction step was repeated until the biomass turned colorless. The extracted pigments were analyzed by Pro-Star 230 high-performance liquid chromatography (HPLC) system equipped with a 2.7 μm particle size, 4.6 × 100 mm, Agilent^®^ Poroshell 120 HPH-C18 column. The mobile phase contained solvent A (acetonitrile/H_2_O, 9:1, v/v) and a mixture of methanol/isopropanol (3:2, v/v) as solvent B. Pigments were eluted at a flow rate of 0.80 ml/min with the gradient parameter, 100% solvent A for 10 min, a gradient increase of the proportion of solvent B to 50% for 5 min, maintain 50% solvent A and 50% solvent B for 20 min, and a gradient decrease of the proportion of solvent B to 0% for the last 10 min. The absorption spectra of the pigments were scanned between 250 and 700 nm. Carotenoids were identified by their absorption spectra and their typical retention times. The standard of each carotenoid was obtained from Sigma-Aldrich (St. Louis, MO, United States).

### Quantification of Glucose Consumption and Citric Acid Secretion

One milliliter of cell culture was centrifuged at 13,000 rpm. The supernatant was collected to determine residual glucose in the medium and the content of secreted citric acid. Glucose was analyzed by using Pro-Star 230 HPLC system equipped with an Aminex HPX-87H ion exclusion column connected with a refractive index (RI) detector. The column was maintained at 65°C. The mobile phase was 0.005 M of sulfuric acid with a flow rate of 0.6 ml/min. The citric acid was analyzed in the same way by HPLC, but it was detected and quantified by an ultraviolet (UV) detector. The cell growth was quantified by measuring the OD_600_ of the culture and dry cell weight (DCW).

## Results

### Lycopene and β-Carotene Production by Expressing *crtEBI* and *carRP* Genes

**FIGURE 2:**
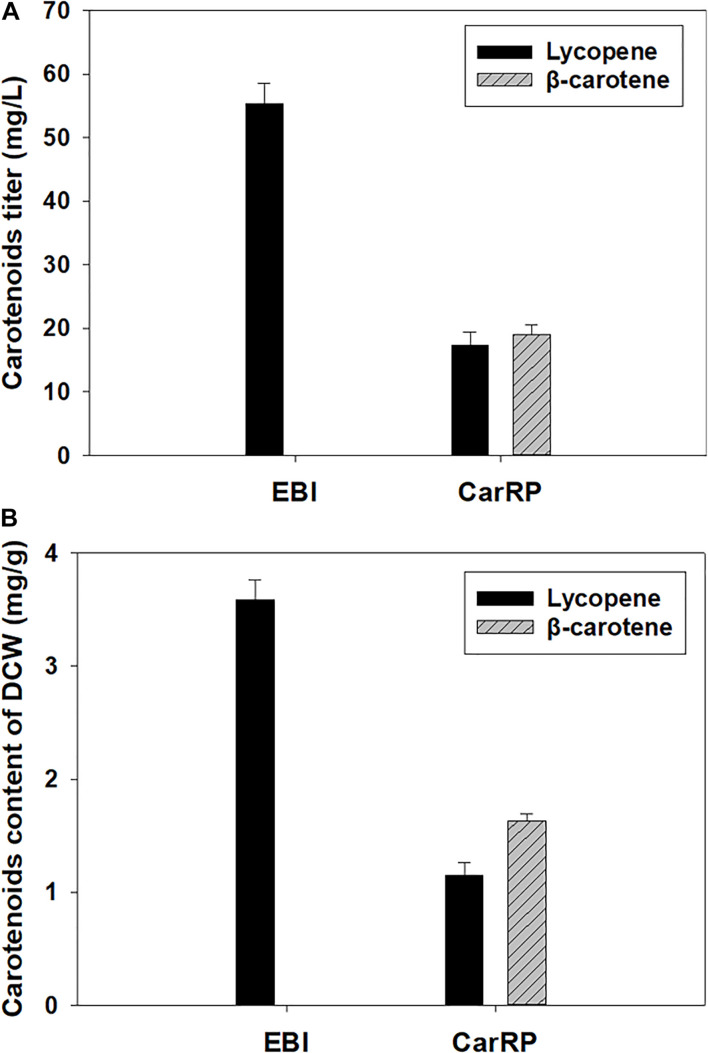
Production of lycopene and β-carotene by the recombinants of *Y. lipolytica*. The strain *Y. lipolytica* EBI was developed by integrating the expression cassettes of three genes, including *crtE*, *crtB*, and *crtI*, into the yeast genome, and *Y. lipolytica* CarRP was further developed by introducing the *carRP* gene. Both strains were grown in yeast extract peptone dextrose (YPD) media containing 50 g/L glucose for 5 days at 28°C and a shaking speed of 200 rpm. The carotenoid titers (mg/L) of each *Y. lipolytica* transformant were displayed **(A)**, and the carotenoid contents (mg/g) of the dry cell weight (DCW) for each *Y. lipolytica* strain were presented **(B)**.

As shown in Figure 2A, the lycopene titer reached 55.35 ± 2.85 mg/L with a lycopene content at 3.59 ± 0.15 mg/g of DCW by the strain *Y. lipolytica* EBI after 5 days of cultivation in a YPD medium containing 50 g/L glucose. To further enable the strain to biosynthesize β-carotene, the gene *carRP* was introduced into the lycopene-producing strain in this study (Velayos et al., 2000). As shown in Figure 2B, *Y. lipolytica* recombinant expressing *carRP* could produce β-carotene. The lycopene and β-carotene titers reached 17.28 ± 1.90 and 11.77 ± 1.16 mg/L by the resultant strain *Y. lipolytica* CarRP, respectively. The lycopene and β-carotene contents were 1.15 ± 0.10 and 0.78 ± 0.05 mg/g based on DCW. Compared with the lycopene-producing strain *Y. lipolytica* EBI, the lycopene titer decreased by 68.78%, and the lycopene content of DCW dropped 67.97% after introducing the *carRP* gene. The total carotenoid titer decreased by 47.52%. Similarly, the total carotenoid content of DCW dropped by 46.24%.

### Zeaxanthin Production by Recombinants Bearing Three Different *crtZ* Genes

**FIGURE 3:**
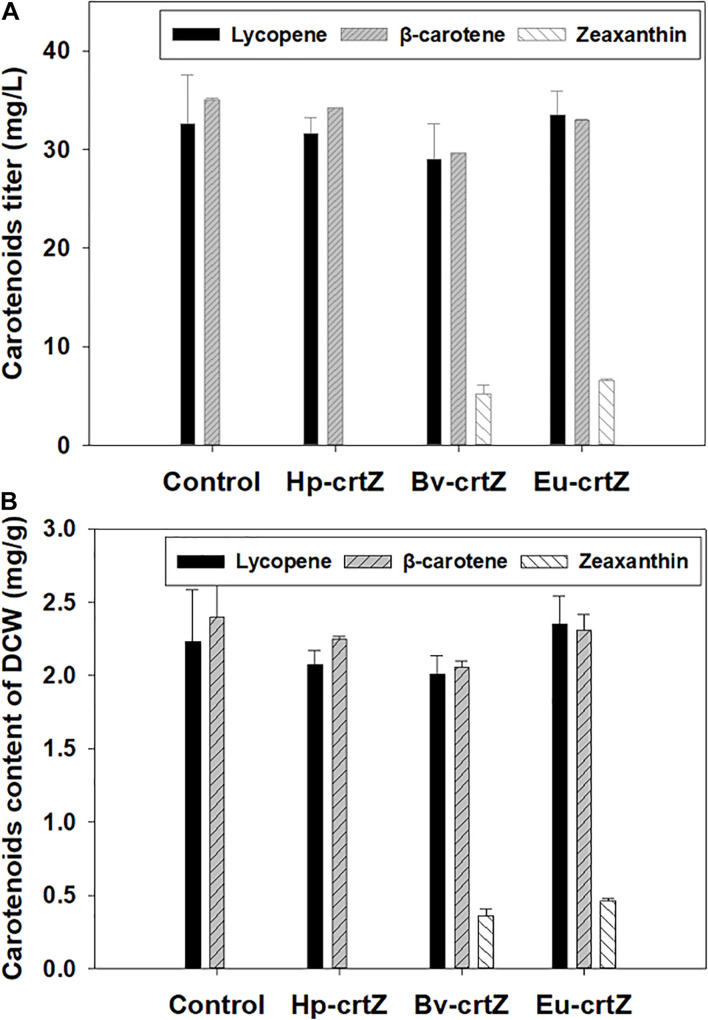
Carotenoids production by *Y. lipolytica* bearing the different *crtZ* genes. The zeaxanthin-producing strains were constructed by the individual transformation of the expression vectors of *Hp-crtZ*, *Eu-crtZ*, and *Bv-crtZ*. The β-carotene producing strain *Y. lipolytica* CarRP was cultured under the same conditions as a control. The strains were grown in yeast nitrogen base (YNB) media containing 50 g/L glucose for 5 days at 28°C and a shaking speed of 200 rpm. The carotenoid titers (mg/L) of the recombinants **(A)** and the carotenoid contents (mg/g, DCW) **(B)** were shown.

To allow the strain to produce zeaxanthin, three different *crtZ* genes were expressed by using the episome expression vector, pJN44, and the developed plasmids were transformed into *Y. lipolytica* CarRP individually. The recombinants bearing the different *crtZ* genes were cultured in YNB media containing 50 g/L glucose for 5 days. As shown in Figure 3A, neither the control strain nor the strain expressing *Hp-crtZ* could produce zeaxanthin, but zeaxanthin could be produced by the β-carotene-producing strain expressing the bacterial genes, *Eu-crtZ* and *Bv-crtZ*. The titers of zeaxanthin production by the strains bearing *Eu-crtZ* and *Bv-crtZ* reached 6.55 ± 0.13 and 5.60 ± 1.90 mg/L, respectively (Figure 3A). The zeaxanthin content of the strains bearing *Eu-crtZ* and *Bv-crtZ* reached 0.46 ± 0.02 and 0.39 ± 0.01 mg/g of DCW, respectively (Figure 3B). The zeaxanthin titer of the recombinant strain expressing *Eu-crtZ* was 18.93% higher than the value of the transformant bearing *BV-crtZ*. Similarly, the zeaxanthin content of recombinant harboring *Eu-crtZ* achieved a 17.75% increase over the value of the *Bv-crtZ* transformant. It indicated that the *Eu-crtZ* from the bacterium *P. ananatis* was an ideal gene for zeaxanthin production among these three *crtZ* genes. In addition to zeaxanthin accumulation, the strains bearing *Hp-crtZ*, *Bv-crtZ*, *Eu-crtZ*, and control did not significantly differ in lycopene production (Figure 3). The lycopene titers of strains bearing *Hp-crtZ*, *Bv-crtZ*, *Eu-crtZ*, and the control were 31.61 ± 1.33, 29.02 ± 2.93, 33.55 ± 1.97, and 32.57 ± 4.05 mg/L, respectively. The lycopene contents were 2.08 ± 0.07, 2.01 ± 0.10, 2.35 ± 0.16, and 2.23 ± 0.29 mg/g of DCW, respectively, in the same order. Besides, the β-carotene titer and content of these strains, including the control strain, were at the same level. The β-carotene titers of the strains bearing *Hp-crtZ*, *Bv-crtZ*, *Eu-crtZ*, and control reached 34.23 ± 1.87, 29.64 ± 1.08, 32.93 ± 0.16, and 34.99 ± 3.30 mg/L, respectively. The β-carotene contents were 2.25 ± 0.02, 2.05 ± 0.03, 2.31 ± 0.09, and 2.40 ± 0.22 mg/g of DCW, respectively, in the same order.

### Improvement of Zeaxanthin Production by Multiple-Copy Integration of *Eu-crtZ*

**FIGURE 4:**
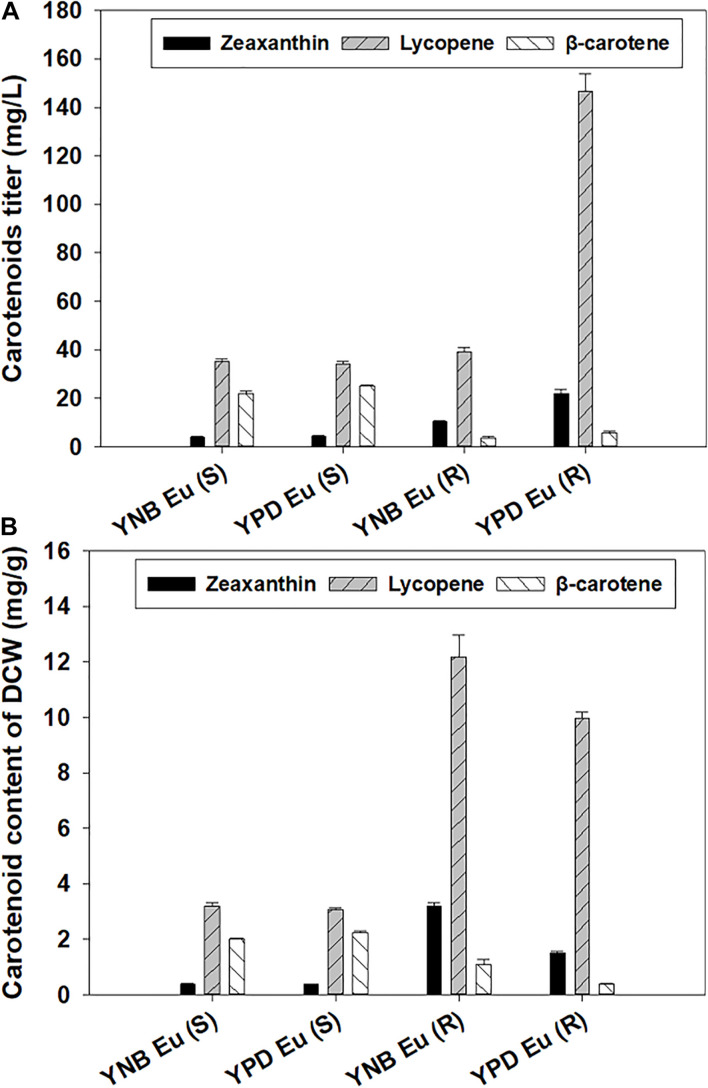
Carotenoids including zeaxanthin production by *Y. lipolytica* integrated with a single- or multiple-copy *Eu-crtZ* gene. The transformant bearing multiple copies of *Eu-crtZ* was designated as Eu (R), and Eu (S) was developed by incorporating a single copy of *Eu-crtZ*. Both strains were grown in YPD or YNB media containing 50 g/L glucose for 5 days at 28°C and a shaking speed of 200 rpm. The carotenoid titers (mg/L) of the recombinants **(A)** and the carotenoid contents (mg/g, DCW) **(B)** were shown.

The gene *Eu-crtZ* was selected for optimizing zeaxanthin production because the expression of this gene led to the highest titer and content for producing the target molecule. Enhancing zeaxanthin production in *Y. lipolytica* was achieved by integrating *Eu-crtZ* gene expression cassette into 26S rDNA region. To clarify the two *Eu-crtZ* recombinants, the transformant bearing *Eu-crtZ* in 26S rDNA region was named for Eu (R), and the *Eu-crtZ* incorporated into the genome with a single copy was designated as Eu (S). The transformants were cultured in YPD and YNB media [named as YPD-Eu (R/S) and YNB-Eu(R/S)] for 5 days for the accumulation of zeaxanthin. As shown in Figure 4A, the zeaxanthin titers reached 21.98 ± 1.80, 10.32 ± 0.42, 6.55 ± 0.13, and 4.38 ± 0.16 mg/L under the different conditions by strains including YPD-Eu (R), YNB-Eu (R), YPD-Eu (S), and YNB-Eu (S), respectively. The zeaxanthin titer of YPD-Eu (R) showed a 4.02-fold, 2.36-fold, and 1.13-fold increase compared with YNB-Eu (S), YPD-Eu (R), and YNB-Eu (S). As shown in Figure 4B, the zeaxanthin content reached 1.49 ± 0.08, 3.20 ± 0.11, 0.46 ± 0.02, and 0.39 ± 0.02 mg/g of DCW under YPD-Eu (R), YNB-Eu (R), YPD-Eu (S), and YNB-Eu (S) conditions, respectively. The zeaxanthin content of YNB-Eu (R) showed a 7.21-fold, 5.96-fold, and 1.14-fold increase compared with YNB-Eu (S), YPD-Eu (S), and YPD-Eu (R). In addition, the strain YPD-Eu (R) showed a 2.24-fold increase than YPD-Eu (S).

For lycopene production, the highest lycopene titer reached 146.53 ± 7.37 mg/L in YPD-Eu (R). The highest lycopene content was 12.18 ± 0.80 mg/g of DCW in YNB-Eu (R). For β-carotene production, the highest β-carotene titer and DCW content reached 25.06 ± 0.35 mg/L and 2.25 ± 0.04 mg/g in YNB-Eu (S). The β-carotene titers reached 5.79 ± 0.59 mg/L (0.39 ± 0.02 mg/g) and 3.50 ± 0.8 mg/L (1.07 ± 0.20 mg/g) in YPD-Eu (R) and YNB-Eu (R), respectively. As shown in Supplementary Figure 2, the zeaxanthin proportion reached 19.46, 12.61, 6.89, and 6.73% of the total carotenoids in YNB-Eu (R), YPD-Eu (R), YNB-Eu (S), and YPD-Eu (S), respectively. The proportion of lycopene in the transformants reached 84.07, 73.94, 53.75, and 57.45% of the total carotenoids in YPD-Eu (R), YNB-Eu (R), YNB-Eu (S), and YPD-Eu (S), respectively. The proportion β-carotene reached 35.83, 39.36, 3.32, and 6.61% of the total carotenoids in YPD-Eu (S), YNB-Eu (S), YPD-Eu (R), and YNB-Eu (R), respectively.

### Zeaxanthin Production, Glucose Consumption, and by-Product Formation

**FIGURE 5:**
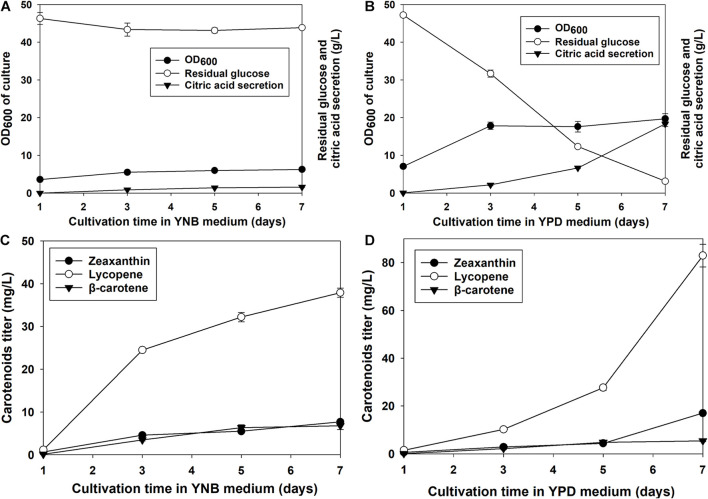
The cell growth, glucose residue content, and citric acid secretion of *Y. lipolytica* EU (R) grown in YNB **(A)** and YPD media **(B)** and the carotenoid production of *Y. lipolytica* EU (R) in YNB **(C)** and YPD media **(D)**. The recombinant *Y. lipolytica* EU (R) was developed by incorporating multiple copies of *Eu-crtZ* into the 26S rDNA region. The strains were grown in YNB or YPD media containing 50 g/L glucose at 28°C and a shaking speed of 200 rpm.

Zeaxanthin production, glucose consumption, and the secretion of citric acid by the recombinant *Y. lipolytica* EU (R) grown in YPD and YNB media were investigated. As indicated in Figure 5, the glucose consumption and the citric acid secretion increased with the cell growth. The cell growth reached the stationary phase at the 3rd day of cultivation. Similarly, the glucose residual and the citric acid remained till the end of the culture. At the end of the culture, the OD_600_ of the culture reached 6.25. Around 43 g/L of glucose remained in the YNB broth, indicating the consumption of 6.95 g/L glucose by the strain (Figure 5A). Besides, 1.57 ± 0.12 g/L of citric acid was produced and secreted in the YNB medium at the end of cultivation. As shown in Figure 5B, the cell growth reached the stationary phase on the 3rd day of cultivation on YPD media. However, the consumption of glucose and the citric acid secretion were observed during the entire cultivation process (Figure 5B). At the end of the culture, the OD_600_ of the yeast culture reached 19.68. Only about 3.1 g/L of glucose remained in the YPD, and the strain consumed 46.89 g/L glucose (Figure 5B). Besides, 18.31 ± 0.70 g/L of citric acid was produced and secreted in the YPD medium at the end of cultivation. We compared the cell growth, glucose consumption, and citric acid secretion in YNB and YPD media. Although the cell growth reached the stationary phase at the same day of cultivation, the OD_600_ of the cell biomass was 2.93 times higher than the value of YNB medium. At the end of cultivation, the cells consumed 5.75 times more glucose in the YPD medium than in the YNB medium. In addition, 10.66 times more citric acid was secreted in the YPD medium than in the YNB medium.

The production of zeaxanthin, lycopene, and β-carotene was illustrated in Figure 5. The carotenoid accumulation of *Y. lipolytica* EU (R) transformants under YNB medium cultivation was shown in Figure 5C. The titers of zeaxanthin and β-carotene increased slowly with the cell growth and cultivation time. The accumulation of lycopene had a significant increase during the 2nd to 3rd day of cultivation. At the end of the culture, the zeaxanthin titer reached 7.69 ± 0.51 mg/L. The titers of lycopene and β-carotene were 37.89 ± 1.08 and 6.79 ± 0.88 mg/L. The carotenoid accumulation of *Y. lipolytica* EU (R) transformants under YPD medium cultivation was shown in Figure 5D. The titers of zeaxanthin and lycopene increased till the end of the culture. The accumulation of lycopene was faster than zeaxanthin. The titers of both carotenoids displayed significant increase on the 5th to 7th day of cultivation. However, the accumulation of β-carotene had a slow increase till the end of culture. At the end of the culture, the zeaxanthin titer reached 17.04 ± 0.27 mg/L. Additionally, the titers of lycopene and β-carotene achieved 82.88 ± 4.72 and 5.45 ± 0.32 mg/L. The *Y. lipolytica* EU (R) transformants produced 1.22 times more zeaxanthin and 1.19 times more lycopene in YPD medium than in YNB medium. However, the β-carotene titers were similar under two medium conditions. The carotenoid yield by using glucose as a substrate can be estimated based on glucose consumption. In the YNB medium, 1.11, 5.45, and 0.97 mg of zeaxanthin, lycopene, and β-carotene were produced by consuming 1 g of glucose (Figure 5). In the YPD medium, 0.36, 1.77, and 0.11 mg of zeaxanthin, lycopene, and β-carotene could be accumulated by consuming 1 g of glucose. Therefore, the *Y. lipolytica* EU transformants cultured in YNB media exhibited higher glucose-to-carotenoid yields than the strain grown in YPD.

## Discussion

Various genes with similar functions from different organisms have been reported for the biosynthesis of zeaxanthin. After the development of β-carotene overproducing *Y. lipolytica*, we first identified the variation of *crt*Z genes for producing the targeted product. Three different CrtZ genes were characterized to produce zeaxanthin in this study. The expression of *Eu-crtZ* gene resulted in the highest zeaxanthin titer and content. However, the expression of *crtZ* from an astaxanthin-producing algal strain, *H. pluvialis*, did not lead to any zeaxanthin accumulation. A similar result was observed in other studies for the engineering of pathways for the biosynthesis of astaxanthin in *S. cerevisiae* (Zhou et al., 2015). It suggested that CrtZ from *Haematococcus* sp. could majorly utilize echinenone and canthaxanthin as the substrate for the biosynthesis of astaxanthin (Zhou et al., 2015). In contrast, CrtZ enzymes from bacteria could catalyze the hydroxylation on β-carotene, β-cryptoxanthin, echinenone, and canthaxanthin for the biosynthesis of zeaxanthin and astaxanthin in engineered microorganisms (Park et al., 2018; Zhang et al., 2020). In this study, the overexpression of *Eu-crtZ* gene resulted in the highest titer and content for zeaxanthin production. After identification of *Eu-crtZ* as an ideal gene for the biosynthesis of zeaxanthin, the gene was integrated into yeast genome to develop a stable recombinant. The use of strong promoters to express the essential genes (Hong et al., 2012) and increasing the copy number of a target gene (Markham et al., 2018) were two primary strategies to improve product production in *Y. lipolytica*. In this study, one of the strongest promoters, P*_*TEF*1*N*_* (Larroude et al., 2018), was used for the expression of the heterologous genes including *crtZ* in *Y. lipolytica*. The integration of *Eu-crtZ* into the rDNA region with multiple copies was carried out to further improve zeaxanthin production. In this study, the discovered *crtZ* gene could serve as a building block for pathway engineering.

The cultivation process is very critical because it links metabolic engineering and product delivery. Cultivation parameters such as culture media, inoculation ratio, and temperature can be optimized to improve product production. Our results showed that the strains cultured in the rich medium YPD could reach higher cell biomass yield and higher carotenoids titers. However, the strains grown in the synthetic culture medium, YNB, achieved a higher content of DCW for zeaxanthin production (Figure 5). These findings were consistent with the results obtained by the metabolic engineering of *Y. lipolytica* for the production of poly(3-hydroxybutyrate) P3HB (Li et al., 2017). These results suggested that the cell growth and product accumulation need to be well balanced to achieve a productive process. As shown in Figure 5B, around 18 g/L of citric acid was detected in the supernatant of the YPD medium. The formation of citric acid as a by-product represented the waste of carbon source for the biosynthesis of the target product and has been observed during lipid accumulation in *Y. lipolytica* under nitrogen-limited culture conditions (Tai and Stephanopoulos, 2013). Both the metabolic engineering approach and cultivation optimization by controlling the dissolved oxygen (DO) level can alleviate the citric acid produced by *Y. lipolytica* to improve target biosynthesis (Sabra et al., 2017).

To develop microbial cell factories for efficiently producing bio-based products, especially natural products, multiple genes usually need to be engineered (Cravens et al., 2019). As shown in Figure 1, it takes more than 10 steps to biosynthesize lycopene from acetyl-CoA and over 14 steps to zeaxanthin. Isoprenoid pathways have been widely engineered in *Y. lipolytica* for producing various natural products (Worland et al., 2020), including carotenoids (Larroude et al., 2018). It is still very challenging to coordinate and balance the expression of these genes to reduce the accumulation of the intermediates but provide sufficient precursors to sustain the target biosynthesis (Zhang et al., 2018). Furthermore, there were variations of enzymes to construct the metabolic pathways. In the present study, the expression of *carRP* from *M. circinelloides* (Velayos et al., 2000) led to the production of β-carotene in *Y. lipolytica* but decreased total carotenoid content. CarRP is a bifunctional enzyme that exhibited the activities of both lycopene cyclase and phytoene synthase. Because *ctrB* encoding phytoene synthase has already been introduced into *Y. lipolytica* for lycopene production, there might be an undesirable intermediate, phytoene accumulated, decreasing overall carotenoid biosynthesis. The MVA pathway for the generation of GGPP precursors has been intensively engineered in *Y. lipolytica* for producing carotenoids and other isoprenoid compounds. Further alleviating the native competition pathway of ergosterol biosynthesis resulted in a higher yield of carotenoids (Arnesen et al., 2020). In the current study, although zeaxanthin has been successfully produced by the metabolic engineering of *Y. lipolytica*, the strain can be further engineered to achieve much higher titer and content by employment of the “push” and “pull” strategies (Tai and Stephanopoulos, 2013).

In addition to pathway engineering, one of the strategies for the improvement of carotenoid accumulation is sequestration of the products in lipid environments (Ma et al., 2019). One of the fascinating features of oleaginous microorganisms such as *Y. lipolytica* is the formation of lipid droplets in the cells. In a microalgal strain *Dunaliella bardawil*, a large amount of β-carotene was stored in lipid droplets (Pick et al., 2019). By building the synergy between lipid accumulation and the formation of lipid-soluble pigments such as lycopene and β-carotene, it resulted in a great improvement of carotenoid production in *Y. lipolytica*. For example, the β-carotene titer reached 6.5 g/L by engineering lipid accumulation and carotenoid formation in *Y. lipolytica* (Larroude et al., 2018). The esterified xanthophylls such as lutein ester could be efficiently stored in lipoprotein particles formed as plastoglobule in marigold (Xie et al., 2021). Similarly, astaxanthin esterified with fatty acids could be sequestered in the lipid droplets of *H. pluvialis* (Pick et al., 2019). With the discovery of the enzymes for esterification of xanthophylls such as xanthophyll acyltransferase (XAT) from bread wheat (*Triticum aestivum*), it paves the way to use lipid droplets as a cellular compartment to promote the sequestration and storage of carotenoids (Watkins et al., 2019). We can capitalize on the uniqueness of *Y. lipolytica* as an oleaginous yeast to further understand and engineer the mechanism underlying carotenoid modification and accumulation with synthetic biology.

## Conclusion

A zeaxanthin biosynthesis pathway was constructed in this study by the expression of *crtE*, *crtB*, *crtI*, *carRP*, and *crtZ* genes in non-carotenoid-producing *Y. lipolytica.* Three different *CrtZ* genes were characterized for zeaxanthin production. *Eu-CrtZ* exhibited the highest zeaxanthin titer and content. The multiple integration of the Eu-CrtZ gene into the yeast genome resulted in a 4.02-fold increase in the titer of zeaxanthin and a 721% increase in the content of zeaxanthin. The optimum zeaxanthin titer and content achieved were 21.98 ± 1.80 mg/L and 3.20 ± 0.11 mg/g in the YPD medium and YNB medium, respectively. Metabolic engineering and fermentation optimization could further increase the zeaxanthin production by *Y. lipolytica*. The results demonstrated the potential of a cell factory for zeaxanthin production.

## Data Availability Statement

The original contributions presented in the study are included in the article/Supplementary Material, further inquiries can be directed to the corresponding author.

## Author Contributions

XX and YX conceived the idea, planned the experiments, and conducted them for the development of yeast recombinants. XX and SC supervised the work. YX collected and analyzed the data. All authors contributed to writing the manuscript and approved the submission of this manuscript.

## Conflict of Interest

The authors declare that the research was conducted in the absence of any commercial or financial relationships that could be construed as a potential conflict of interest.

## Publisher’s Note

All claims expressed in this article are solely those of the authors and do not necessarily represent those of their affiliated organizations, or those of the publisher, the editors and the reviewers. Any product that may be evaluated in this article, or claim that may be made by its manufacturer, is not guaranteed or endorsed by the publisher.
