# Supplementary material for: Metabolic Engineering of Non-carotenoid-Producing Yeast Yarrowia lipolytica for the Biosynthesis of Zeaxanthin
Source: Front Microbiol. 2021 Oct 7;12:699235. doi: 10.3389/fmicb.2021.699235 (PMC8529107; doi:10.3389/fmicb.2021.699235)

Supplementary Material

Metabolic engineering of Non-carotenoid-producing Yeast *Yarrowia lipolytica* for Biosynthesis of Zeaxanthin

Yuxiao Xie, Shulin Chen, Xiaochao Xiong*
Department of Biological Systems Engineering, Washington State University, Pullman, WA 99164-6120, USA

***Correspondence:**
Corresponding Author: Dr. Xiaochao Xiong, E-mail: xcxiong@wsu.edu
Phone: +1 509-335-5996; Fax: +1 509-335-2722

Table S1. Sequences of the primers used in this study. The generated restriction sites were underlined.

Primer	Target gene	Sequence (5'- 3')
CrtE-exp1	*crtE*	CGGCG<u>AAGCTT</u>ATGGATTATAACAGCGCGGAT
CrtE-exp2		TATTAT<u>CCCGGG</u>TCACTGCGCATCCTCAAAG
CrtB-exp1	*crtB*	CGTGT<u>AAGCTT</u>ATGAACAACCCCTCTCTGC
CrtB-exp2		TAATTT<u>CCCGGG</u>TTACAGGGGTCGCTGCCACA
CrtI-exp1	*crtI*	CCCTTA<u>AAGCTT</u>ATGAAGCCCACCACCGTGAT
CrtI-exp2		CGTTAT<u>CCCGGG</u>TTAAATCAGGTCCTCGAGCAT

Figure S1. Schematic map of the plasmid pZX13 for multiple-copy integration into the ribosomal DNA (rDNA) of *Y. lipolytica*. The restriction enzyme, NdeI or ApaI can be used to linearize the plasmid if the cloned gene does not have such a digestion site.
Amp^R, ampicillin resistance; rDNA-up/down: two portions of rDNA; and *ura3*, *Y. lipolytica* auxotrophic selection marker.

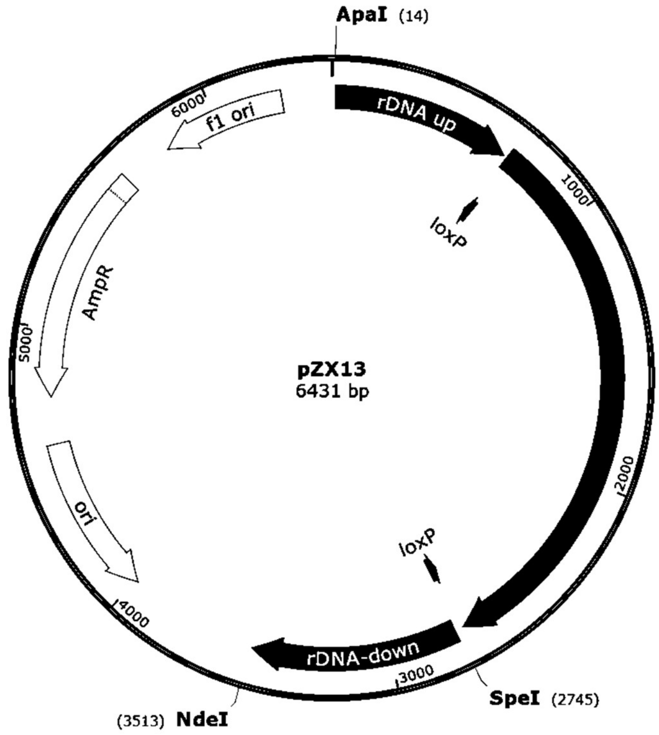

Table S2. DNA sequence of synthesized *carRP* from *Mucor circinelloides*

1	ATGCTGCTGA	CCTACATGGA	GGTCCATCTG	TACTACACCC	TCCCCGTGCT
51	CGGTGTGCTG	TCTTGGCTCT	CCCGACCTTA	TTACACCGCT	ACCGACGCTC
101	TGAAGTTTAA	GTTTCTGACC	CTCGTCGCCT	TCACCACCGC	TTCCGCTTGG
151	GACAACTACA	TCGTCTACCA	CAAGGCTTGG	TCCTACTGCC	CTACTTGCGT
201	CACTGCCGTG	ATCGGTTACG	TGCCTCTGGA	GGAGTACATG	TTCTTCATTA
251	TCATGACTCT	GCTGACCGTG	GCCTTTACTA	ATCTGGTCAT	GCGATGGCAC
301	CTCCACTCCT	TCTTCATCCG	ACCCGAGACC	CCCGTCATGC	AGTCTGTCCT
351	CGTGAGACTC	GTGCCTATCA	CCGCTCTGCT	GATTACCGCC	TACAAGGCTT
401	GGCACCTCGC	TGTCCCCGGT	AAACCCCTCT	TTTACGGTTC	TTGCATTCTG
451	TGGTACGCTT	GCCCCGTGCT	GGCTCTCCTC	TGGTTTGGCG	CCGGCGAGTA
501	CATGATGCGA	CGACCTCTGG	CCGTGCTGGT	GTCTATCGCT	CTGCCTACTC
551	TGTTTCTGTG	TTGGGTCGAC	GTGGTCGCTA	TCGGCGCCGG	TACTTGGGAT
601	ATCTCTCTGG	CCACCTCTAC	TGGCAAATTC	GTCGTCCCCC	ATCTGCCCGT
651	CGAGGAGTTC	ATGTTCTTCG	CTCTGATCAA	CACTGTGCTG	GTCTTCGGCA
701	CTTGCGCCAT	CGACAGAACC	ATGGCCATCC	TCCATCTGTT	CAAGAATAAA
751	TCCCCCTACC	AGCGACCCTA	CCAGCATTCC	AAGTCCTTCC	TCCACCAGAT
801	TCTGGAGATG	ACTTGGGCCT	TTTGTCTCCC	CGACCAAGTG	CTCCACTCCG
851	ATACCTTTCA	TGACCTCTCC	GTCTCTTGGG	ACATCCTCCG	AAAGGCTTCC
901	AAGTCTTTCT	ACACTGCCTC	TGCCGTGTTC	CCCGGCGACG	TCCGACAAGA
951	GCTGGGTGTG	CTGTATGCTT	TCTGCCGAGC	CACCGATGAC	CTCTGCGATA
1001	ACGAGCAAGT	CCCCGTGCAG	ACCCGAAAGG	AACAGCTGAT	TCTGACTCAC
1051	CAGTTCGTGT	CCGATCTGTT	CGGCCAGAAG	ACCTCTGCCC	CTACCGCCAT
1101	CGACTGGGAC	TTTTACAACG	ACCAGCTCCC	CGCCTCTTGC	ATCTCCGCCT
1151	TCAAGTCCTT	CACTCGACTG	CGACACGTGC	TGGAAGCCGG	CGCCATTAAG
1201	GAGCTCCTCG	ATGGCTACAA	GTGGGATCTG	GAGAGACGAT	CTATCAGAGA
1251	CCAAGAGGAT	CTGCGATACT	ACTCTGCTTG	CGTCGCCTCC	TCCGTCGGCG
1301	AGATGTGCAC	CCGAATCATT	CTGGCTCACG	CCGACAAGCC	CGCCTCCAGA
1351	CAGCAGACCC	AGTGGATCAT	CCAGCGAGCT	CGAGAAATGG	GTCTCGTGCT
1401	CCAGTACACC	AACATCGCCC	GAGACATTGT	GACCGACTCC	GAAGAGCTCG
1451	GTCGATGTTA	TCTGCCCCAA	GACTGGCTGA	CCGAGAAGGA	AGTGGCTCTC
1501	ATTCAAGGCG	GTCTCGCTCG	AGAGATTGGT	GAAGAGCGAC	TGCTGTCCCT
1551	CTCTCACCGA	CTCATCTACC	AAGCCGACGA	GCTCATGGTC	GTGGCCAATA
1601	AGGGCATTGA	CAAGCTGCCT	TCTCACTGTC	AAGGCGGTGT	CAGAGCCGCT
1651	TGCAACGTCT	ATGCCTCTAT	TGGCACCAAG	CTCAAGTCCT	ACAAGCACCA
1701	TTACCCCTCC	CGAGCCCACG	TCGGCAACTC	CAAGAGAGTG	GAGATCGCTC
1751	TGCTCTCCGT	CTACAATCTG	TACACCGCTC	CCATCGCTAC	CTCTTCTACT
1801	ACCCACTGCC	GACAAGGCAA	GATGAGAAAT	CTCAACACCA	TCTAA

Table S3. DNA sequence of synthesized *Bv-CrtZ* from *Brevundimonas vesicularis*

```
1       ATGTCGTGGC CCACCATGAT CCTGCTCTTT CTCGCCACTT TCCTGGGAAT
51      GGAGGTCTTC GCCTGGGCTA TGCACCGATA CGTTATGCAT GGTCTGCTCT
101     GGACCTGGCA CCGATCTCAC CATGAGCCCC ATGACGATGT GCTCGAGCGA
151     AACGACCTGT TTGCCGTGGT CTTCGCCGCT CCTGCCATCA TTCTGGTCGC
201     TCTGGGCCTC CACCTGTGGC CTTGGATGCT CCCTATTGGC CTGGGAGTTA
251     CCGCTTACGG CCTGGTGTAC TTCTTTTTCC ACGATGGCCT GGTTCATCGA
301     CGATTTCCTA CCGGTATCGC CGGCCGATCC GCTTTCTGGA CTCGACGAAT
351     TCAGGCCCAC CGACTCCACC ATGCTGTCCG AACTCGAGAG GGATGCGTTT
401     CTTTTGGTTT CCTGTGGGTG CGATCCGCTC GAGCTCTCAA GGCTGAGCTG
451     TCGCAGAAGC GAGGCTCTTC CTCGAACGGA GCTTAA
```

Table S4. DNA sequence of synthesized *Eu-crtZ* from *Pantoea ananatis* (formerly *Erwinia uredovora*)

1	ATGCTGTGGA	TCTGGAACGC	CCTGATCGTG	TTCGTGACCG	TGATCGGCAT
51	GGAAGTGATC	GCCGCTCTGG	CCCACAAGTA	CATCATGCAC	GGCTGGGGCT
101	GGGGATGGCA	CCTGTCTCAC	CACGAGCCTC	GAAAGGGCGC	CTTCGAGGTG
151	AACGACCTGT	ACGCCGTGGT	GTTCGCTGCC	CTGTCTATCC	TGCTGATCTA
201	CCTGGGCTCT	ACCGGCATGT	GGCCCCTGCA	GTGGATCGGA	GCCGGCATGA
251	CCGCCTACGG	CCTGCTGTAC	TTCATGGTGC	ACGACGGCCT	GGTCCACCAG
301	AGATGGCCCT	TCCGGTACAT	TCCCCGAAAG	GGCTACCTGA	AGCGACTGTA
351	CATGGCCCAC	CGAATGCACC	ACGCCGTGCG	AGGCAAGGAA	GGCTGCGTTT
401	CTTTCGGCTT	CCTGTACGCA	CCTCCTCTGT	CTAAGCTGCA	GGCTACCCTG
451	CGAGAGCGAC	ACGGCGCTCG	AGCCGGCGCT	GCCAGAGATG	CCCAAGGCGG
501	CGAGGACGAG	CCCGCCTCTG	GCAAGTAA		

Table S5. DNA sequence of synthesized *Hp-crtZ* from *Haematococcus lacustris*

1	ATGCTGTCCA	AGCTCCAGTC	TATCTCTGTC	AAGGCCCGAC	GAGTGGAGCT
51	CGCTCGAGAC	ATCACCCGAC	CCAAGGTCTG	CCTCCACGCT	CAGCGATGTT
101	CTCTGGTGCG	ACTGCGAGTG	GCTGCTCCTC	AGACCGAAGA	GGCTGTCGGT
151	ACTCAGCAAG	CCGCTGGCGC	TGGCGATGAG	CACTCTGCCG	ACGTGGCCCT
201	CCAGCAACTC	GACCGAGCTA	TTGCCGAGCG	ACGAGCCCGA	CGAAAGCGAG
251	AGCAACTGTC	CTACCAAGCC	GCTGCCATTG	CCGCCTCCAT	TGGCGTCTCC
301	GGCATTGCCA	TCTTCGCCAC	CTATCTGCGA	TTCGCCATGC	ACATGACCGT
351	CGGTGGCGCT	GTCCCTTGGG	GTGAAGTGGC	CGGTACTCTG	CTGCTGGTGG
401	TGGGTGGTGC	TCTGGGCATG	GAGATGTACG	CTAGATACGC	CCACAAGGCC
451	ATCTGGCACG	AATCCCCTCT	CGGTTGGCTG	CTCCACAAGT	CCCACCACAC
501	CCCTCGAACC	GGTCCCTTCG	AGGCTAACGA	TCTGTTCGCC	ATCATTAACG
551	GCCTCCCCGC	TATGCTGCTG	TGCACCTTTG	GTTTCTGGCT	GCCCAACGTG
601	CTCGGCACCG	CTTGTTTCGG	CGCTGGTCTG	GGCATCACTC	TGTACGGCAT
651	GGCCTACATG	TTCGTCCACG	ACGGTCTCGT	GCACAGACGA	TTCCCCACCG
701	GCCCCATTGC	TGGTCTGCCT	TACATGAAGC	GACTGACCGT	CGCCCACCAG
751	CTGCACCACT	CTGGTAAGTA	CGGCGGCGCC	CCTTGGGGTA	TGTTTCTCGG
801	TCCCCAAGAG	CTCCAGCACA	TCCCCGGCGC	CGCTGAGGAA	GTGGAGCGAC
851	TGGTCCTCGA	ACTGGATTGG	TCCAAGCGAT	AA	

Figure S2. Carotenoid profiles of *Y. lipolytica* strains under different culture conditions. The transformant bearing multiple copies of *Eu-crtZ* was designated as Eu (R), and strain Eu (S) was developed by incorporating single copy of *Eu-crtZ*. Both strains were grown in YPD or YNB media containing 50 g/L glucose for five days at 28 ºC and a shaking speed of 200 rpm. The contents of lycopene, β-carotene, and zeaxanthin produced by the recombinants were measured by HPLC, and the portion was shown as percentage of total carotenoids.

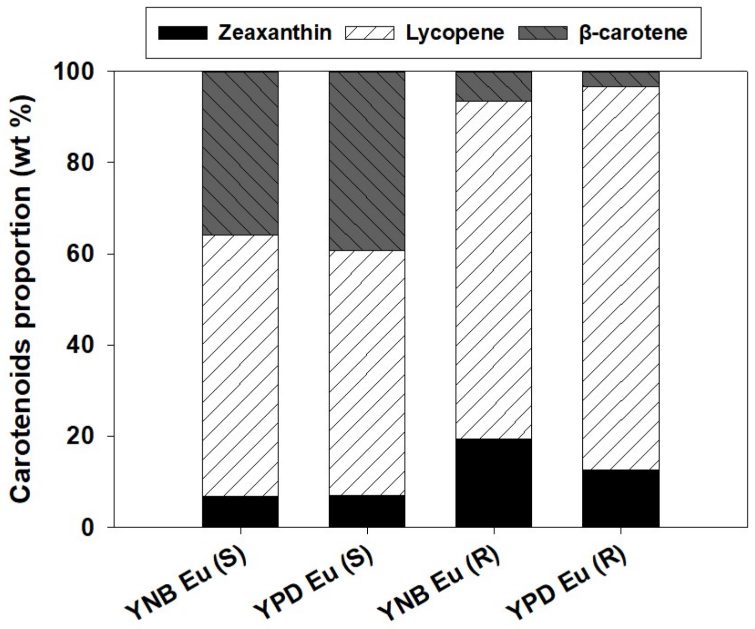

Supplement: Supplementary file 1 [file Data_Sheet_1.PDF]
